# Macroscale multimodal imaging reveals ancient painting production technology and the vogue in Greco-Roman Egypt

**DOI:** 10.1038/s41598-017-15743-5

**Published:** 2017-11-14

**Authors:** John K. Delaney, Kathryn A. Dooley, Roxanne Radpour, Ioanna Kakoulli

**Affiliations:** 1 National Gallery of Art, 6th and Constitution Avenue NW, Washington, D.C. 20001 USA; 2 Materials Science and Engineering Department, University of California Los Angeles, BOX 951595, Engineering V, 410 Westwood Plaza, Los Angeles, CA 90095-1595 USA; 3 Department of Materials Science and Engineering (DMSE), Massachusetts Institute of Technology (MIT), Room 6-113, 77 Massachusetts Ave, Cambridge, Massachusetts 02139 USA

## Abstract

Macroscale multimodal chemical imaging combining hyperspectral diffuse reflectance (400–2500 nm), luminescence (400–1000 nm), and X-ray fluorescence (XRF, 2 to 25 keV) data, is uniquely equipped for noninvasive characterization of heterogeneous complex systems such as paintings. Here we present the first application of multimodal chemical imaging to analyze the production technology of an 1,800-year-old painting and one of the oldest surviving encaustic (“burned in”) paintings in the world. Co-registration of the data cubes from these three hyperspectral imaging modalities enabled the comparison of reflectance, luminescence, and XRF spectra at each pixel in the image for the entire painting. By comparing the molecular and elemental spectral signatures at each pixel, this fusion of the data allowed for a more thorough identification and mapping of the painting’s constituent organic and inorganic materials, revealing key information on the selection of raw materials, production sequence and the fashion aesthetics and chemical arts practiced in Egypt in the second century AD.

## Introduction

Advances in technology and miniaturization of chemical imaging technologies – adapted mainly from airborne scanners – including reflectance hyperspectral imaging (HSI) in the visible and shortwave infrared region (VSWIR, ~400 to 2500 nm) and scanning macroscale X-ray fluorescence (MA-XRF) spectroscopy^1–7^, have enabled their deployment in the field and in museums, making enormous strides for *in situ* noninvasive characterization and analysis of important works of art ranging from Old Masters’ and modern paintings, to wall paintings and polychrome archaeological artifacts. Diffuse reflectance HSI in the VSWIR region provides information about molecular structure of inorganic and organic materials based on electronic and vibrational transitions (overtones and combination bands). Luminescence HSI (400–1000 nm) offers complementary information on the molecules and more specifically the intrinsic luminophores in the materials analyzed, based on their characteristic light emission (luminescence) after the absorption of photons initiated by photoexcitation at specific wavelengths. MA-XRF (2 to 25 keV) contributes with hyperspectral data (elemental distribution images), elemental information with photon emissions of characteristic X-rays. For the analysis of ancient paintings, multimodal imaging spectroscopy offers unparalleled potential in identification of both organic and inorganic materials, hitherto impossible without sampling and microanalysis. It further enables the mapping of both molecular and elemental data for every pixel in the image across the entire surface of the painting, thus aiding in accurate attributions and interpretations and informing on production technology and raw materials selection.

**Figure 1:**
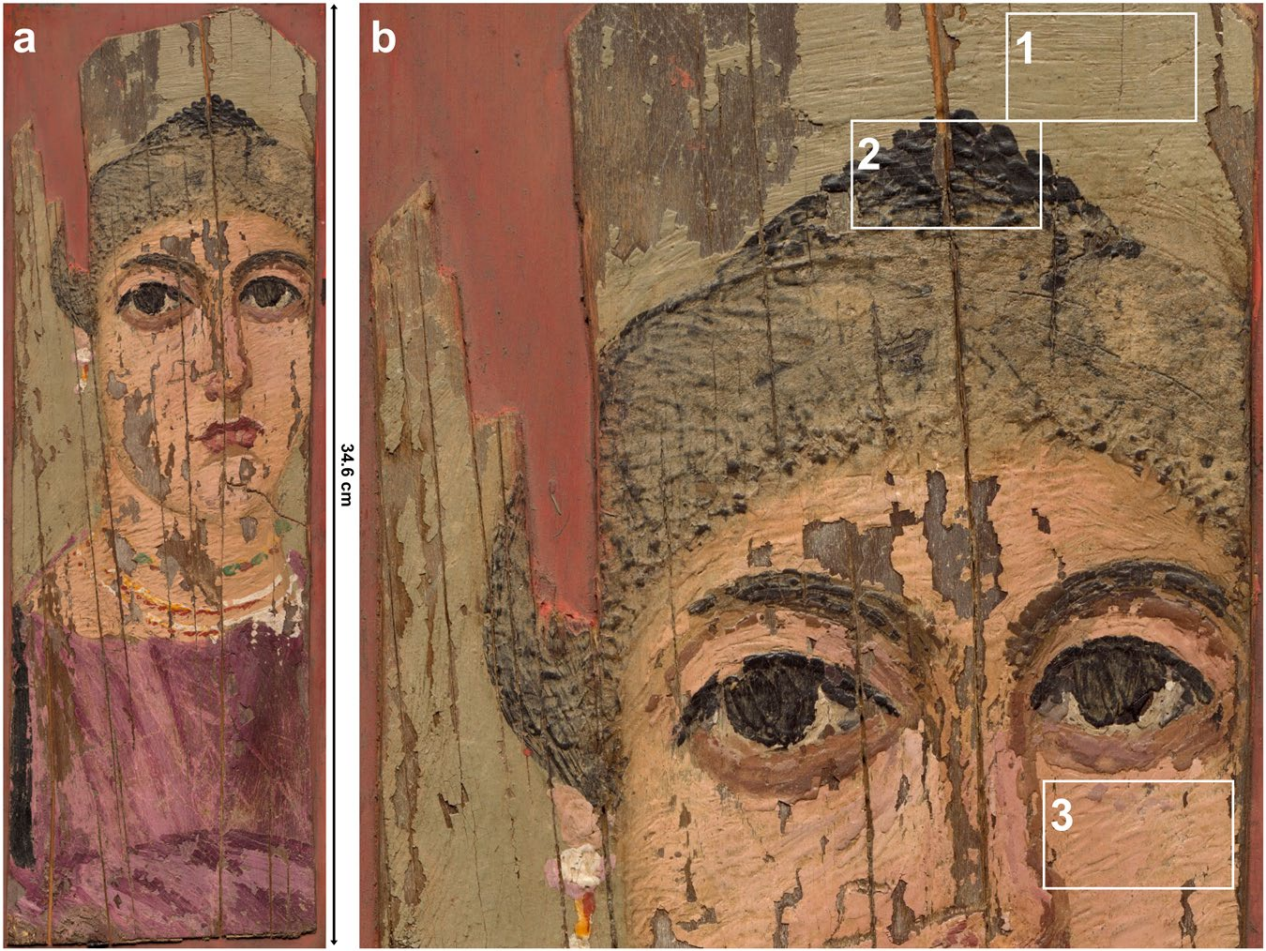
(**a**) Color image of the Fayum painting “Portrait of a woman”, c. 2^nd^ century AD, accession number 1956.12.1, gift of Lewis Einstein, in the collection of the National Gallery of Art, Washington DC, depicting a female figure from a noble family as conveyed by the hairstyle and jewelry (dimensions: 34.6 × 11.5 cm). *Courtesy National Gallery of Art*, *Washington*; (**b**) Detail of the painting, illustrating different application methods of the heated wax-based paint resulting in distinct surface topography, most likely using three different tools as described by Pliny^11^: a fine painter’s brush or *penicillus* (1), a *cauterium* (a metal spoon or hollowed spatula) (2), and a *cestrum* (a type of engraver) (3).

Here we demonstrate the potential of multimodal chemical imaging spectroscopy with results from the fusion of the three data cubes obtained from diffuse reflectance and luminescence HSI and MA-XRF spectral imaging modalities, employed for the analysis of a complex and archaeologically significant Greco-Roman painting (“Portrait of a Woman”) of the second century AD, from the collection of the National Gallery of Art in Washington DC (Fig. 1). The data cubes from all three hyperspectral imaging modalities were co-registered, or spatially aligned to one another, which enabled the comparison of reflectance, fluorescence, and XRF spectra at each pixel in the image for the entire painting. This fusion of the data, or the comparison of the three different types of spectra for a given pixel, allowed for a more thorough identification and mapping of the painting materials. This is the first application of multimodal imaging spectroscopy on an archaeological panel painting over eighteen hundred years old, abetting fingerprint identification of the organic and inorganic constituent materials and the *chaîne opératoire* – the operation sequence by which raw materials were selected, processed, and transformed into a cultural object.

Greco-Roman portraits are also known as ‘Fayum portraits’ or ‘Romano-Egyptian portraits’, and they are the oldest surviving portrait-paintings, mainly crafted on wooden supports^8–10^. It is believed to depict images of real people who lived in Egypt during the Ptolemaic (323–30 BC) and the Roman rule (30 BC – fourth century AD). The style is typical of the fashion developed during the Roman Empire in Egypt from the middle of the first century BC to the early fourth century AD reflecting the Greek culture, education and civic life.

The panel painting under consideration is a funerary portrait of a noble woman (Fig. 1). Her high socioeconomic status is denoted by 1) the elegant hairstyle dating to the period of the emperor Trajan of the second century AD; the purple tunic with *clavus* (a decorative stripe adorning the garment) and extensive jewelry and 2) the exceptional artistry and painterly execution of the painting with a highly-textured surface achieved by mixing the pigments in the binding medium, the blending and juxtaposition of the paint, and the application of the paint using different tools and methods (Fig. 1b(1–3)). The woman depicted in this painting is framed by a yellow/green-yellow background, dressed in a purple tunic with a dark *clavus* on the right (proper) side. The tunic exhibits different shades of purple/red-purple to express shading, indicating a high level of skill in the painting technique. The jewelry consists of a pendant earring and three necklaces, inlaid with what appear to be pearls and gemstones.

**Figure 2:**
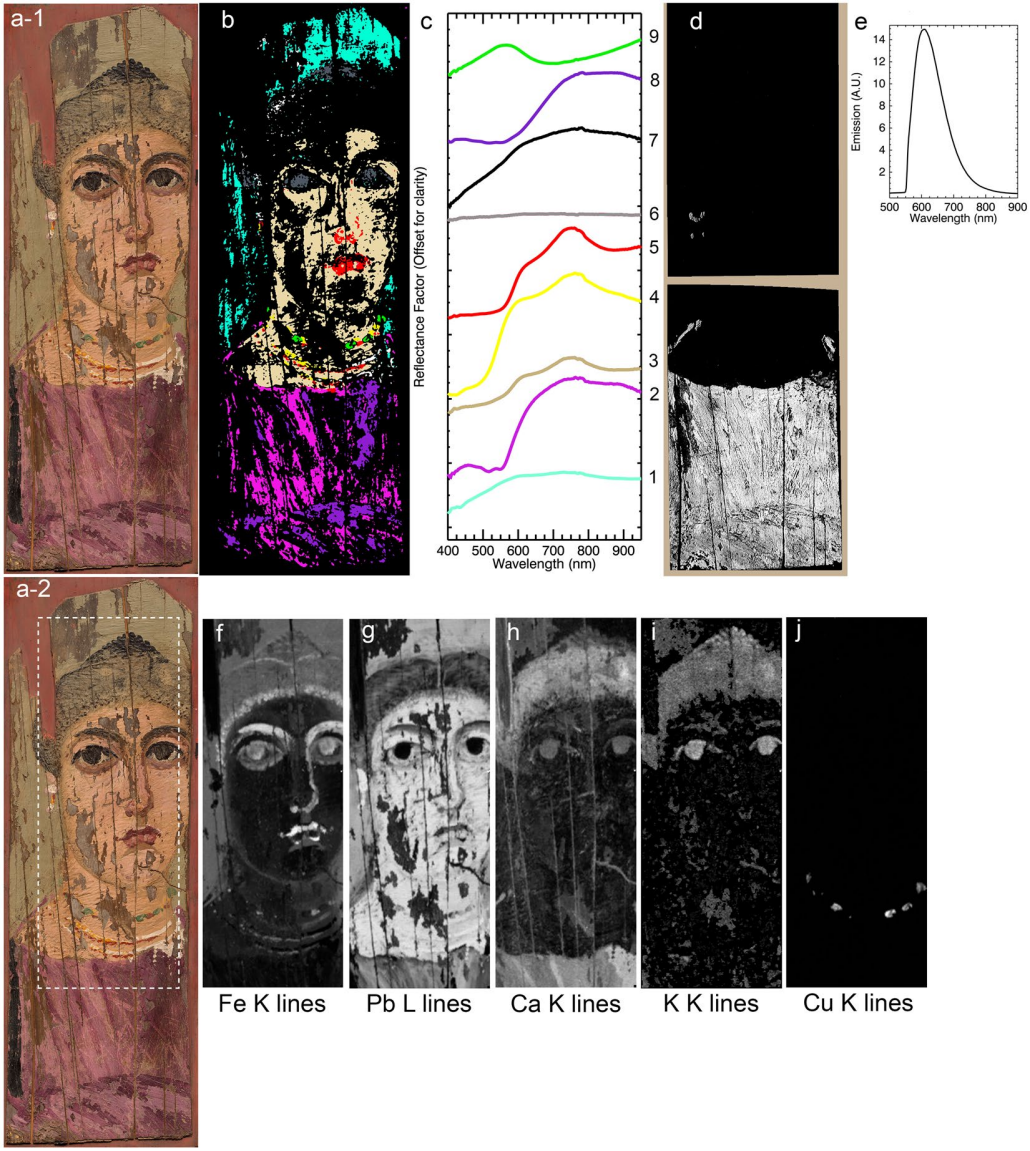
Calibrated reflectance, luminescence and XRF image data cubes that have been separately registered to a reference color image. (**a-1** and **a-2**) Color images of the portrait; (**b**) Map of the reflectance spectral endmembers (**c**), ‘black’ in the map are unassigned pixels and spectral endmember# 7 shown in black maps to the “white map” areas; (**d**) Map of the luminescence spectral endmember (**e**) after the correction of the luminescence spectral cube for self-absorption; (**f**–**j**) corresponding XRF elemental maps, of the sum of the K or L lines, for the area marked with a white rectangle in **a-2**, for: Fe (**f**), Pb (**g**), Ca (**h**), K (**i**) and Cu (**j**).

**Figure 3:**
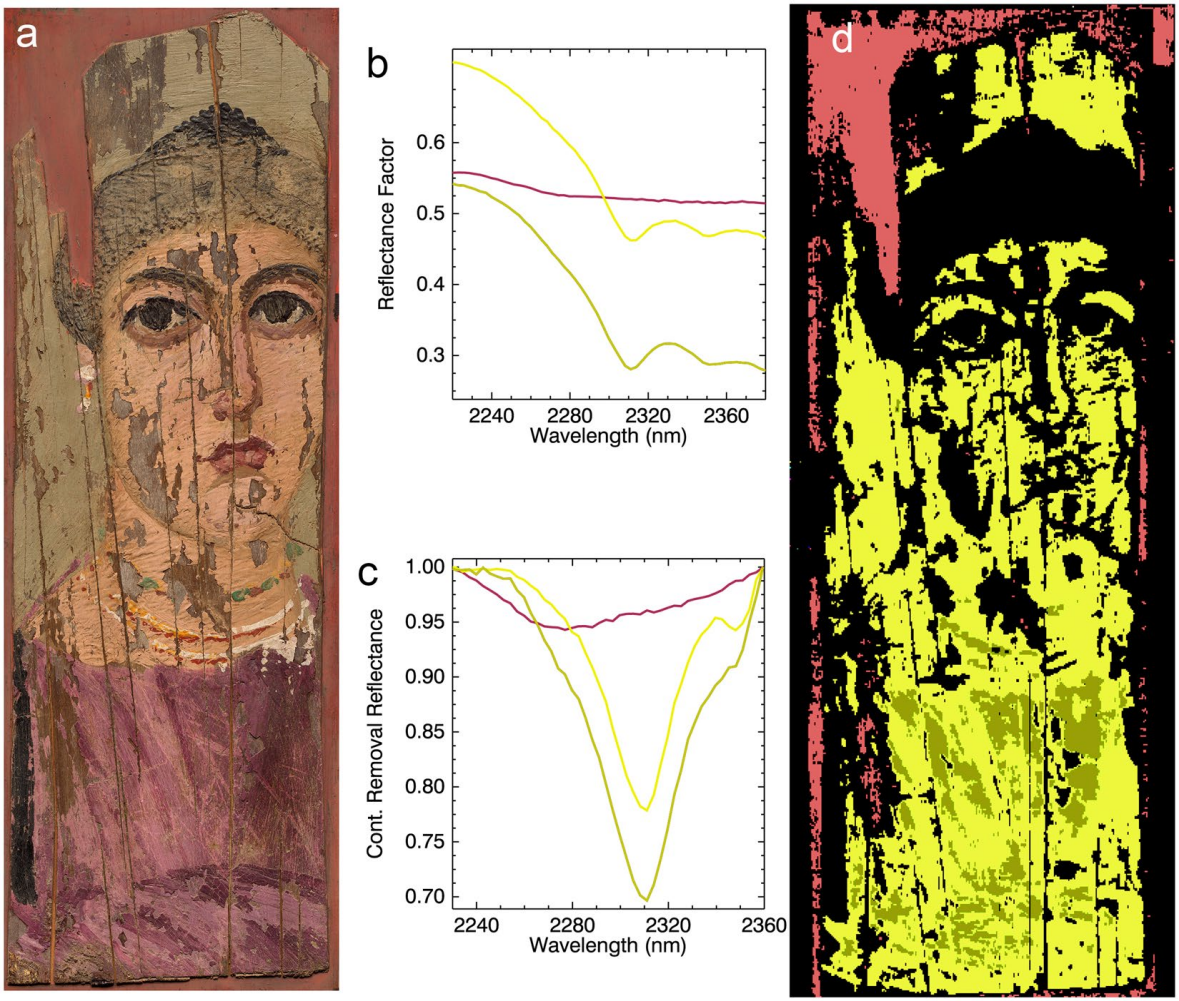
(**a**) Color image of the portrait; (**b**) reflectance spectral endmembers; (**c**) continuum removed endmembers which were used in the mapping; (**d**) chemical map of spectral signatures from endmembers in Fig. 3c.

Reflectance and luminescence 3-D data cubes of the painting, with spatial information represented in the X-Y plane and spectral data in the Z-direction, were collected using low noise, high light sensitivity, pushbroom line-scanning hyperspectral cameras (see Methods). Once calibrated, these data cubes were spatially registered to a reference color image using a novel algorithm that automatically finds control point pairs before applying the necessary transformation to achieve sub-pixel accuracy (Fig. 2) ^12^. Since the recorded hyperspectral reflectance and luminescence 3-D data cubes collectively contain over 1.2 M spectra, multivariate statistical analysis based on convex geometry was employed to find a basis set of reflectance and luminescence spectra, or spectral endmembers, that best describe the variance of these data sets (see Methods). Spectral endmembers defining reflectance and/or luminescence vectors, representing signatures of the different materials present in the painting, were used to produce distribution maps of the pigments or pigment mixtures (Fig. 2b–e) as well as the paint binding medium (Fig. 3) across the surface of the painting. MA-XRF data cubes were collected with a raster scanning system, and elemental maps were constructed from fitting of the emission lines to obtain the integrated peak areas of characteristic X-ray emissions (Fig. 2f–j).

The application of multimodal imaging spectroscopy combining reflectance, verified by spot analysis using fiber optic reflectance spectroscopy (FORS), luminescence, and X-ray fluorescence HSI datasets, provided new insights in the technology and processes involved in the production of this ancient painting – an intriguing and complex multilayered system. Findings from the analysis of the paint binding medium, pigments and production technology of the portrait are presented in the broader context of the chemical arts of the period, considering ancient literary sources, alchemical manuals, and depictions on surviving material culture representing painters at work, tools and instruments used in painting, sculpture, alchemy and other related ancient industries. By embedding the results of the painting materials (pigments and binder) in the framework of material selection and operational processes, we have further interrogated the extent to which materials technologies reflect the socioeconomic, civic and political environment in which Greco-Roman painting was developed and entrenched.

## Results and Discussion

For the characterization of organic materials used as paint binding media, such as lipids, carbohydrates and proteinaceous materials, spectral bands in the mid-infrared (mid-IR) related to fundamental vibrational transitions are typically used. However, the associated overtones and combination bands in the infrared between 1600 and 2400 nm have also been successfully utilized to identify some of the organic materials of these classes. Using this spectral region, it has been shown that paint binding media such as drying oils, alkyds, protein-based glues, and egg yolk tempera^13–15^ in paintings can be identified and mapped. For lipid-rich binders, diagnostic absorptions include a combination band of asymmetric/symmetric stretching and bending (ν + δ) of the methylenic (CH_2_) groups with a characteristic doublet at 2300 to 2312 nm depending on lipid type (drying oil, egg yolk, wax) and 2346 nm, respectively, and the first overtone (2ν) of CH_2_ stretching that occurs as a doublet near 1723 and 1755 nm^14^.

Spectral endmember extraction from HSI reflectance datasets gave a discriminating absorption feature at 2311 nm indicative of wax (C-H stretch and bend combination, known to occur between 2311 to 2312 nm), as opposed to drying oil (2302 to 2304 nm) or egg yolk (2307 to 2309 nm). Thus the 2230 to 2360 nm spectral region was used for the mapping of wax (Fig. 3). The abundance and widespread distribution of wax throughout the painting, except for areas of paint loss or areas having high concentration of black pigments (infrared absorbing (see below)), combined with the distinctive textured surface of the paint layer, indicate the use of melted beeswax as the binding medium mixed with pigments to create a paste-like paint.

The technique of using melted beeswax as the binder is also widely known as “encaustic”, from the Greek “εγκαυστική” [enkavsteké] meaning to heat or burn in (Greek “ἐγκαίειν” [enkaíein]). Pliny the Elder’s Natural History provides the earliest written record of the encaustic style of painting. As he remarks, no one knows who the inventor of “painting in wax” was, but according to Pliny, the most famous painter of this technique was Pausias of Sicyon, a celebrated painter of the mid-fourth century BC^11^.

Observations of the surface using raking light showed variability in the roughness of the surface, pointing to different methods of application of the paint for the background, the flesh tones, the hair and the garments. Close examination of the surface topography suggests the use of different tools for the application of the melted-wax paint, most likely employing a *penicillus* (painter’s fine-hair brush); a *cauterium* (metal spoon or hollowed spatula that could be heated and used in encaustic painting for burning in the wax), or a *cestrum* (engraver tool) (Fig. 1) as implied by Pliny^11^ and other ancient authors in reference to the encaustic technique. Illustrations suggesting the use of heated wax-based paints and the tools used for this ‘burn in’ technique can be seen in surviving ancient iconography depicting artists at work. Notable examples include: a) the painting decorating the interior of the Kerch sarcophagus^16,17^ of the second century AD, showing what has been accepted as a painter in his studio working on a picture in encaustic, for he is heating his painting tool (cestrum?) or possibly even the wax pigment in a charcoal brazier; and b) a fourth century BC Apulian terracotta krater^18^ at the Metropolitan Museum of Art collection, portraying an artist painting a lion-skin on a white marble statue of Hercules. Next to the sculpture, an assistant of the artist is heating the paint in a basin of coals, suggesting encaustic technique.

These representations also reflect the importance of color and the deployment of colored finishes on surfaces during this period. Artists during the Greek and Roman period, employed a variety of coloring compounds either pure or in mixtures to achieve different hues and tonalities. These include a wide spectrum of natural materials and synthetic products: iron oxides, hydroxides, sulfates and silicates; calcium carbonates and phosphates; lead and/or arsenic-containing inorganic compounds; copper-based organometallic complexes; dyes of plant- or animal-origin, mordanted with inorganic translucent substances, such as potash alum to form lake pigments; and polycrystalline sintered materials^19–24^.

Sample- and label-free characterization of the pigments and pigment mixtures in the Fayum portrait was achieved with macroscale multimodal chemical imaging, which offered an improved diagnostic reliability due to the complementary nature of the retrieved information. The success of such fusion requires accurate image registrations between the different image modalities. Analysis of the spectral features of the reflectance endmembers combined with elemental information from the XRF imaging provided unbiased characterization of the pigments (Table 1). The assigned pigments include the presence of the natural minerals: natrojarosite, goethite, hematite, calcium carbonate; the synthetic inorganic compound: lead white; the synthetic organic pigment: charcoal black; and the synthetic organic-inorganic hybrids: copper-carboxylate (an organo-copper compound) and madder lake (a hydroxyanthraquinone (HAG)-metal chelate). No blue pigments such as Egyptian blue or indigo, commonly encountered in Greco-Roman portraiture and other contemporary paintings and polychrome artifacts, were identified in this painting^20,25,26^.

**Table 1:** Pigment identification.

Reflectance Endmember (Fig. 2c)	Reflectance Endmember Absorption Features	Luminescence Endmember (Fig. 2e)	Luminescence Emission max	MA-XRF Element(s)	Pigment(s) assignment	Color	Location
9	~695 nm and UV-violet region; and maximum reflectance in the blue-green region			Cu	*Organo-copper	Emerald Green	Necklace
8	sub-bands: ~514 nm & ~549 nm (weak)	+ +	604 nm	Ca	*Madder Lake, Calcium Carbonate	Purple/Red-Purple	Tunic
7	~1445 nm			Pb	*Lead White	White	Necklace, Earring and light tones of other hues
6	< 5% reflectance			K, Ca, Fe	*Charcoal Black (Vine Black)	Black	Hair, Eyes, and dark tones of other hues
5	~550 nm (shortward), ~620 nm (shoulder), ~850 nm			Fe	*Hematite	Red	Necklace
5	~550 nm (shortward), ~620 nm (shoulder), ~850 nm, ~1445 nm			Fe, Pb	*Hematite, *Lead White, Charcoal Black	Purple/Red-Purple	Lips
5	~550 nm (shortward), ~620 nm (shoulder), ~850 nm			Fe	*Hematite, *Charcoal Black	Brown	Shadows in flesh/face
4	~475 nm (shortward), ~920 nm			Fe	*Goethite	Yellow	Necklace
3	~435 nm, ~660 nm (shoulder), ~870 nm, ~1445 nm, ~1840, ~1940 nm			Fe, Pb	*Goethite, Natrojarosite, *Hematite, *Lead white	Red-Orange (light tone)	Flesh tone
2	sub-bands: ~514 nm & ~549 nm	+ +	604 nm	Ca	*Madder Lake, *Calcium Carbonate	Red-Purple	Tunic
1	~435 nm (sharp), ~650 nm (shoulder), ~910 nm, ~1445 nm, ~1840 nm, ~1923 nm			Fe, Pb	*Natrojarosite, *Lead White, Charcoal Black	Yellow/Green-Yellow	Background

Note 1: The [*] indicates pigment with major contribution in the hue; the [++] indicates luminescence endmember spectrum in Fig. 2e.

The choice of using a pure pigment or pigment mixtures combined with the binding medium produced various colored paints: green (emerald green identified in the jewelry/necklace), yellow/green-yellow (background), yellow (mainly identified in jewelry/necklace), light tone of red-orange (flesh tone), red (jewelry/necklace), purple/red-purple (garments/tunic, lips), red-purple (garments/tunic) and brown (shadows in flesh). The selection of specific pigments to depict distinct components of the painting, such as the garments or the green gems, suggests knowledge of dye preparation and dyeing processes to color textiles and alchemical practices to imitate semi-precious stones through the coloring of stone crystals. Beyond the influence of the pigments selection, the final aesthetics in the painting in terms of physical and optical attributes depended on a variety of factors, mainly the painting technique used, as well as the pictorial means employed such as *chiaroscuro* (Greek “σκιαγραφία” [skiagrafia]). In this painting, *chiaroscuro* achieved by the mixing of white and/or black in the paint was used to portray depth and volume. The choices of pigments and the treatment of the paint in this portrait, suggest a level of artistry and advanced technique for paintings of this age.

**Figure 4:**
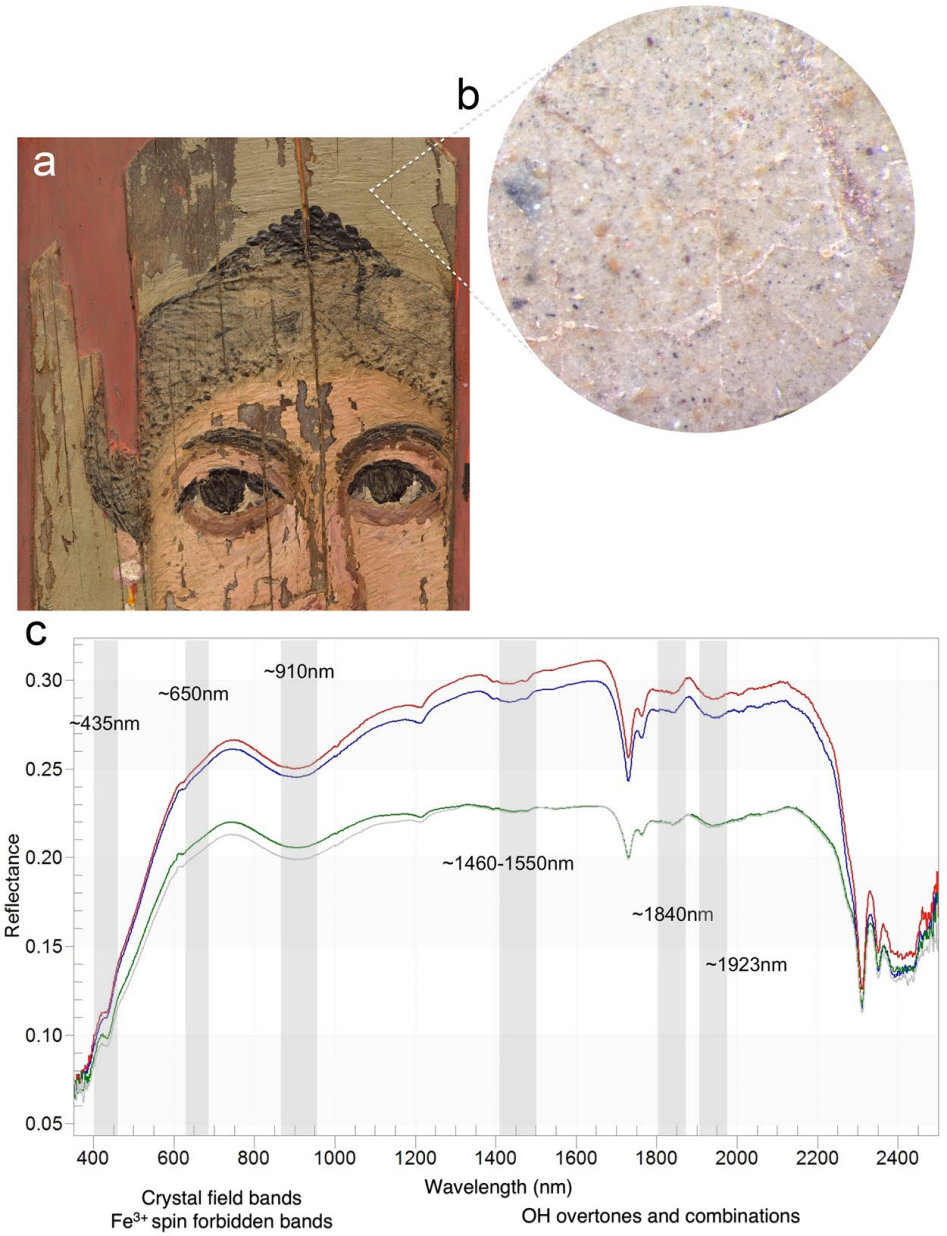
(**a**) Detail of the painting pointing to an area under magnification (seen in **b**); (**b**) Photomicrograph of the yellow/green-yellow background showing dispersed yellow-brown and small black particles. (**c**) Reflectance spectra from four areas in the background with characteristic features indicative of natrojarosite in a wax binder (features at 2311, 2352 nm).

Endmember extraction from the reflectance HSI cube (Fig. 2c) and verified by FORS spot analysis (Fig. 4) gave spectra (endmembers #1, 3) with characteristic absorptions at ~432 nm (corresponding to 6A1g to 4Eg, 4A1g transition of a single Fe^3+^ center), a shoulder at ~630 nm and a broad absorption around 910 nm, caused by ligand field effects (Fe^3+^ spin forbidden bands). In addition, the FORS spectra show the NIR overtones and combinations of ν and δ OH-associated absorption bands near 1465–1550 nm and 1840 nm, respectively, and a series of possible S–O related bands in the 2200–2500 nm region^27,28^. These spectral features led to the unbiased identification of natrojarosite, a sodium iron sulfate (Na^+^Fe^3+^_3_[(SO_4_)_2_(OH)_6_). Jarosites ((K^+^, Na^+^)Fe^3+^_3_ (SO_4_)_2_(OH)_6_) are natural minerals belonging to the alunite family where the Fe^3+^ is octahedrally-coordinated. These natural minerals are common weathering products of oxidized iron sulfides under acidic conditions and they often form jointly with ferric oxyhydroxides.

Precise identification of natrojarosite (sodium-substitutions) in this painting was established through the diagnostic absorption bands shifting in wavelength position, owing to the different sizes of the two cations Na and K with radii of 1.39 and 1.64 Å and charge/radius values of 0.65 and 0.56, respectively^28,29^, impacting in different degrees their local electronic environments. In this painting, natrojarosite was identified primarily in the yellow/green-yellow background in combination with charcoal black (the region where endmember #1 maps, Fig. 2c), and in the flesh tones (the region where endmember #3 maps) mixed with lead white, hematite and to a lesser extent goethite. In addition, the absence of K in the MA-XRF mapping (characteristic of the K-jarosite), further supports the presence of the Na > K jarosite mineral; Na could not be detected/mapped by MA-XRF, due to instrumental limitations in the detection of low Z elements (below magnesium with Z = 12). Na > K jarosites with yellow, brown and red hues were extensively used as pigments in Hellenistic and Roman paintings in the Eastern Mediterranean^19,30^.

MA-XRF mapping of characteristic X-ray emissions of Fe associated with the yellow necklace and the flesh paint, combined with corresponding reflectance HSI distribution of signatures from endmembers and FORS spectra with absorptions at ~475 nm and 920 nm, determined the presence of goethite (α-FeOOH), an iron-bearing hydroxide mineral. Its red counterpart, hematite (Fe_2_O_3_), is identified by its characteristic strong absorption shortward of ~550 nm, a shoulder at ~620 nm and a broad absorption around ~850 nm^31^. Hematite is the major component in the flesh paint, the brown shading and contour of the hair, and the red-purple color of the lips. The distinctive Fe^3+^ ligand field transitions in goethite (resulting in absorption near ~475 nm) helped distinguish it from natrojarosite^28,32^ (absorption ~435 nm) and hematite (absorption ~550 nm). The absence of manganese indicates that no umbers (Fe_2_O_3_/MnO_2_) were used, thus confirming that the brown hues were obtained by mixing ferric oxyhydroxides with other pigments.

The classical authors Theophrastus (fourth century BC)^33^, Vitruvius (first century BC)^34^ and Pliny (first century AD)^11^ all discuss natural ferric oxide (hematite) pigments. They highly praise the ‘sinoper’ red from Anatolia but make reference of other deposits in Egypt, the Balearis islands and Lemnos in the Aegean^34^. Pliny^11^ also refers to two blends of red ochre: the first, calcined with lead white to form a red-orange mixture called *sandyx* to be used for the treatment of flesh tones and the second, mixed with the *sandyx* and used as an undercoating or modeling layer. *Sandyx*, a blend of red ochre and lead white was indeed identified in this painting as the major color for the flesh.

**Figure 5:**
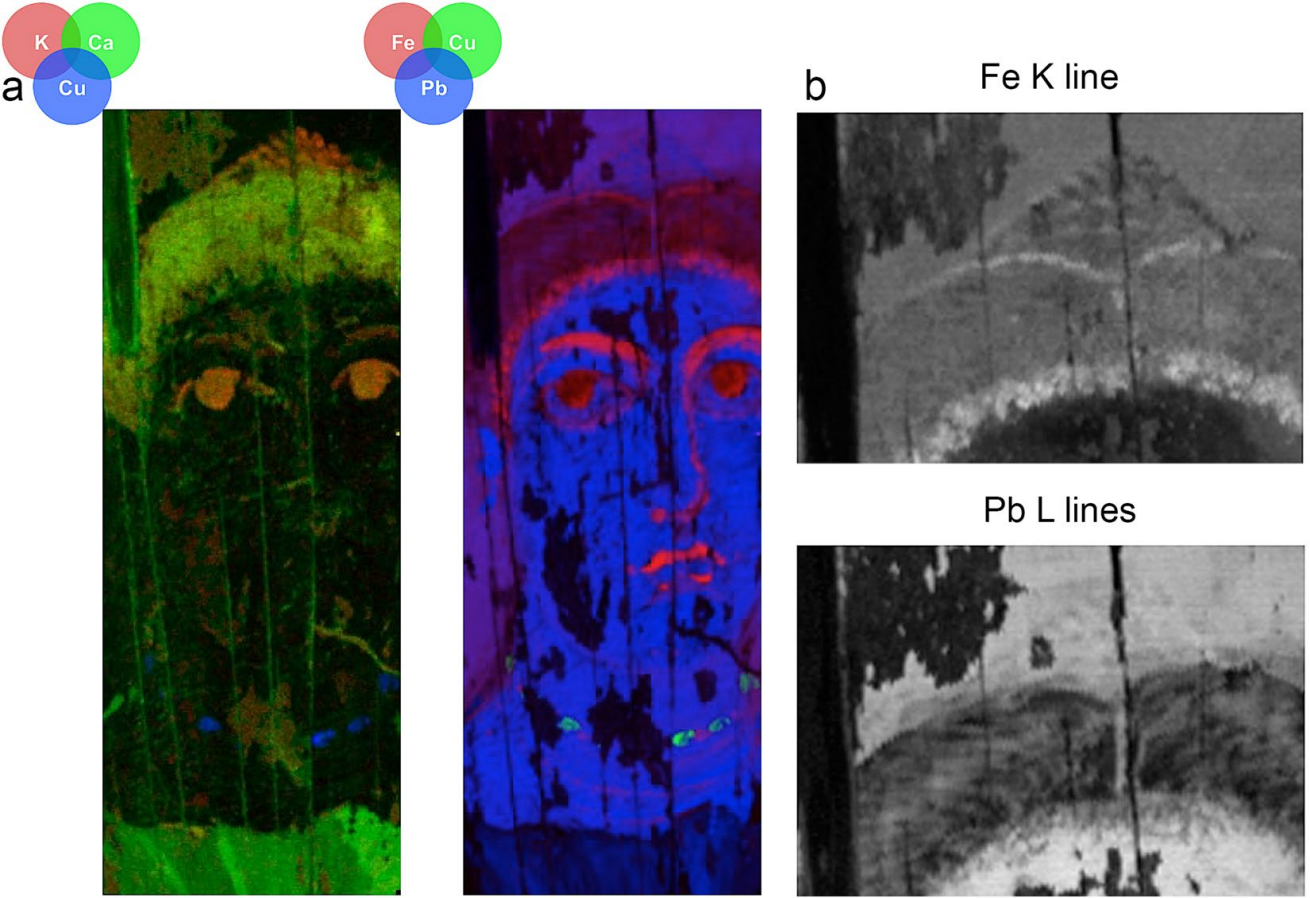
(**a**) MA-XRF composite element maps and (**b**) details of single element maps of sum of alpha and beta characteristic X-ray emissions of elements. The details of the single element maps (**b**) show hair braids that had been painted out.

The elemental mapping of Fe mainly in the hair area achieved by MA-XRF, revealed features in the painting not discernible by the naked eye or the reflectance hyperspectral mapping owing to the low reflectance values. More specifically, these maps exposed details on the fashion of the hairstyle and the operation sequence for the build-up of the painting and the underdrawings or foundation colors defining the contours and forms (Fig. 5). The original hairstyle, as delineated in the Fe map, shows the central hair parting with a bun at the top of the head and possibly two hair braids wrapped around the bun, a typical coiffure of the period.

The map of the characteristic X-rays (Lα) emitted by Pb indicates the extended use of a lead-based pigment as the principal constituent of the white paint in the jewelry, as the body paint in the flesh mixed with natrojarosite and ferric oxyhydroxides, and in the yellow/green-yellow background. Corresponding data from reflectance HSI endmembers showed the distinctive absorption band ~1445 nm, diagnostic of lead white (not shown), an artificial pigment containing hydrocerussite (Pb_3_(CO_3_)_2_(OH)_2_) and cerussite (PbCO_3_) at different proportions, although the 1445 nm feature is ascribed to the –OH group and thus only hydrocerussite^35^. The ancient writers Theophrastus, Pliny the Elder and Vitruvius, all describe its preparation from metallic lead and vinegar leading to an insoluble substance, washed by decantation^11,33,34^. Though contemporary alchemical texts do not provide any recipe how to synthesize lead white, in a description on “the coloration of silver”, one of the ingredients mentioned is *cerussa* which is the Latin name for the pigment. Dioscorides^36^ refers to it as *psimithion* “ψιμύθιον”, and describes a similar preparation for the product to that of Theophrastus^33,37–39^. He further suggests grinding it into a fine powder and sifting it, so that it becomes a very fine-grained powder to be used as eye medication. In an archaeological context, lead white was found in Hellenistic and Roman paintings as a pigment^19,35,40^ and in a powder form^35^, associated with female burials, which is suggestive that it was used as a cosmetic^41^.

Spatial distribution maps of photon emissions characteristic of Cu Kα energy, identified its presence localized only within the dark green painted regions associated with the depiction of green gemstones or rock crystal-inlays in the necklace (Fig. 2j). Complementary data from reflectance HSI endmembers and FORS, showed a characteristic broad absorption at ~695 nm and also in the ultraviolet (UV)-violet region and a reflection in the blue-green, suggesting an organometallic copper compound such as a copper-carboxylate complex. These features distinguish it from verdigris (Cu(O_2_CCH_3_)_2_) and malachite (Cu_2_CO_3_(OH)_2_) which have absorption maxima at 720 nm and 800 nm respectively.

The association of copper-carboxylate with the green ‘gemstones’ in the jewelry draws parallels to alchemical accounts^42^, providing detailed recipes on how to stain rock crystals with copper-coloring compounds, analogous to the ones used in the painting, in imitation of emerald and other gems. To produce this pigment, a copper salt like copper acetate (verdigris), was most likely mixed with heated beeswax to transform into Cu(II)-carboxylate (Equation 1).

Equation 1. Synthesis of copper carboxylate from copper acetate.

$$\begin{array}{c}Cu{({O}_{2}CC{H}_{3})}_{2}\mathop{\longrightarrow }\limits^{heated\,beeswax}Cu{({O}_{2}CR)}_{2}\\ {\rm{Copper}}\,{\rm{acetate}}\,\,\,\,\,\,\,\,\,\,\,\,\,\,\,\,\,\,\,\,{\rm{Cu}}({\rm{II}})-\mathrm{carboxylate}\end{array}$$

Theophrastus provides a recipe for the preparation of verdigris where the copper plates are covered by sour grape skins^33^. A different method for preparing verdigris, specifically for emerald substitutions, is given in detail in the *Graecus Holmiensis* alchemical papyrus also known as the Stockholm papyrus of the third century AD. In this recipe, copper plates are suspended over acetic acid vapor in a well-enclosed container. According to the papyrus, green gemstones were imitated by applying this copper salt (verdigris) and organic coloring materials to quartz after its surface had been roughened. The numerous recipes for simulating gems further suggest that these artificially colored rock crystals were in high demand and frequently used by the ancients.

**Figure 6:**
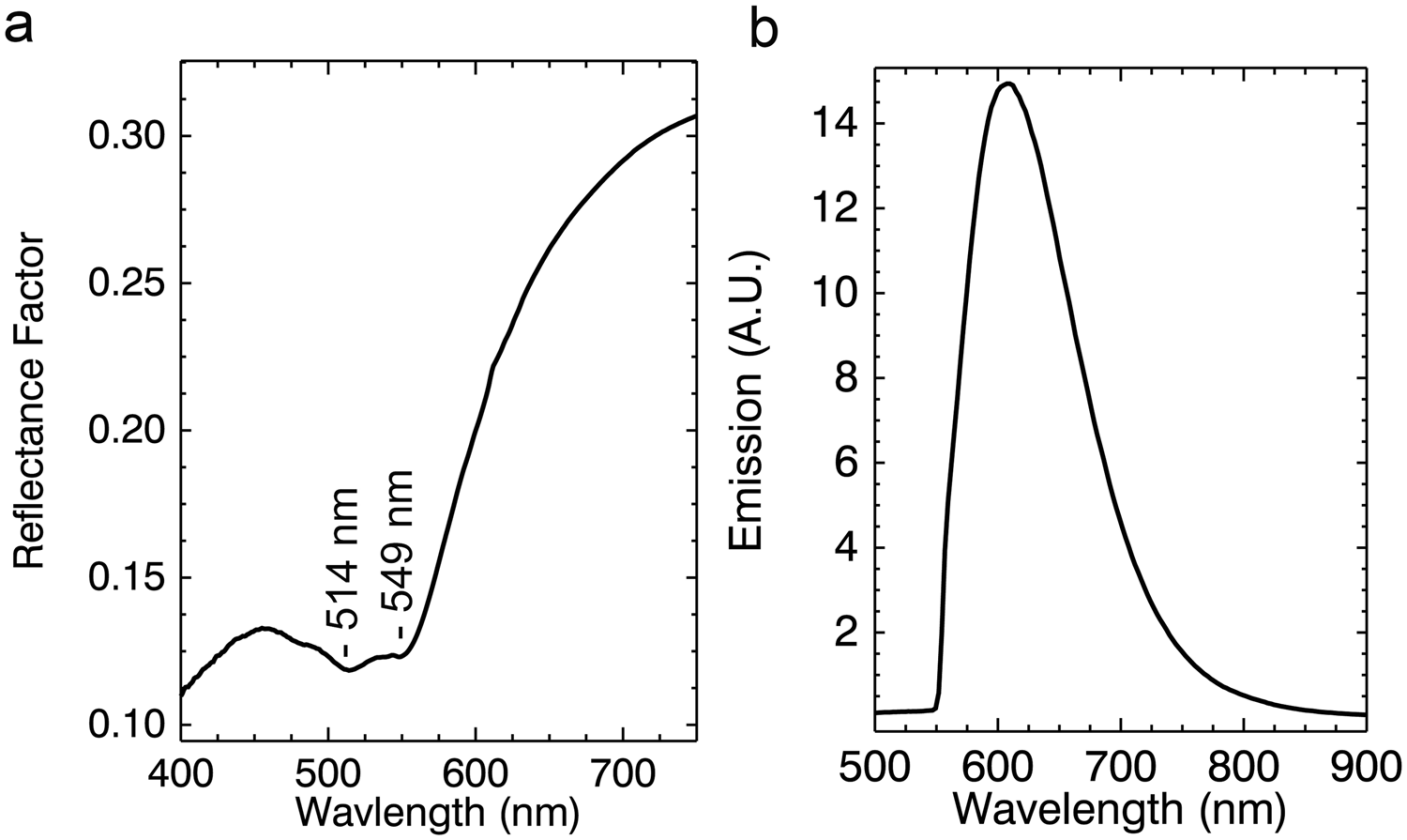
(**a**) Reflectance spectrum from the red-purple tunic showing absorption features associated with π-π* transition of madder and (**b**) emission spectral endmember used for the luminescence map in Fig. 2d.

The purple tunic is described by two reflectance endmembers, #2 and #8 representing the dark and light areas (Fig. 2c). Reflectance endmember spectrum #2, shown again in Fig. 6a, has a strong absorption with two sub-bands at ~510–520 nm and ~540–550 nm^43–45^ consistent with a madder lake. In the deeper purple regions in the tunic represented by endmember #8 these two sub-bands are not readily apparent. However, after correction of the luminescence hyperspectral image cube for self-absorption, the analysis of the luminescence emission cube yielded one endmember (Figs 2e and 6b). The luminescence maximum emission at ~600–610 nm is characteristic of madder lake prepared from direct extracts of the roots of the plant and not through secondary extraction from previously madder-dyed textiles^46^. This luminescence endmember maps to the entirety of the purple tunic (same regions as the reflectance endmembers #2 and #8) and also in the pendant earring.

Madder is a natural dyestuff found in the roots of flowering plants of the genus Rubia belonging to the Rubiaceae family. It is the oldest known and most widely used red colorant in the ancient world to dye textiles and to prepare the translucent pigment known as madder lake. While there are several different species of madder plants, two seem to have predominated the Hellenistic and Roman world: the wild species known as a Rubia Peregrina and the cultivated species, Rubia Tinctoria. Their discrimination has been based on the ratio between the two prevalent hydroxyanthraquinones (HAQ) in the wild versus the cultivated species: alizarin (1,2-dihydroxyanthraquinone) and purpurin (1,2,4-trihydroxyanthraquinone).

The third century Stockholm papyrus^42^ (alchemical manual) gives numerous recipes to dye textiles using madder, in a process that is similar to the preparation of a madder lake pigment. The production of a lake pigment requires the precipitation of a dyestuff extract onto an inorganic substrate through the reaction of the dye molecules with a metal ion to form an ion-dye chelate (organometallic complex). Potash alum (potassium aluminum sulfate – KAl(SO_4_)_2_·12H_2_O) is the most typical substrate used in both dye and pigment production. To enable chelation of the dyestuff molecule to the aluminum ion, an alkaline salt or an alkaline earth metal, such as calcium carbonate or hydroxide, was commonly employed^19,47^. The reaction that occurs between the metal ion and alkali then precipitates with the dyestuff.

The choice of using a mordanted red dyestuff, particularly madder, as the pigment to color the garments of the noble woman depicted in the portrait in red-purple tones, provides another paradigm to infer social context and contemporary practices in Egypt during the Greco-Roman period. Production technology and raw materials selection cannot be looked at in isolation from the societies that produced them. This informed choice of using madder lake as the pigment to color the garments in the painting suggests knowledge of the use of this pigment as the primary colorant to dye textiles and the operational sequences for its production and application. The detailed descriptions of how to produce this colorant and its function as a textile colorant in the alchemical papyri further indicate the connections among the various chemical arts and associations to everyday life.

Calcium (Ca) and potassium (K) detected by MA-XRF mainly in the black of the eyes, eyebrows and hair may be associated with the use of wood ash during the partial burning of plants for the preparation of charcoal black. Ca could further denote a source of calcium carbonate that may have been used for the chelation of the organic dyestuff in the preparation of the red lake^19,48,49^.

## Conclusions

The Hellenistic and Roman era in Egypt was a period of experimentation and innovation where technology was strongly embedded in social practices and scientific and philosophical pursuits of a growing curiosity about the physical word. The art of portrait painting in Greco-Roman Egypt represented people of the Hellenized communities, following the Greek settlement in Egypt at the time of Ptolemy, the Macedonian general who founded the Ptolemaic Dynasty in 331 BC. During this period, Egypt, with Alexandria as its center, had become the nexus of a new intellectual universe. Portraiture, like other artistic expressions, moved from the conventionalized earlier representations, to a Hellenistic approach which introduced stylistic and technical innovations such as shading, translucency and three-dimensionality. As demonstrated by archaeological evidence, chemically analogous synthetic materials were found in different contexts, signifying their different functions: e.g. lead white and madder lake, two of the most prominent pigments in the Hellenized world, were found as powders or organic salts (pastes or emulsions) in a preparatory stage to be used as paints or cosmetics and medicinal substances^35,50–52^. The production of these pigments – as new compounds – with specific properties to render desirable aesthetic effects in painting involve different chemical procedures such as mixing, firing, sublimation and decantation^19,53^. These processes are entangled and interrelated to the manufacture of materials for other industries and further illustrates the close affinities between the various ‘chemical arts’ such as mining, metallurgy, dyeing, and fermentation. The cultural and socio-political milieu in Egypt and elsewhere in the empire during the Hellenistic and Roman period, with philosophies driving experimentation, influenced material choices and processes involved for the production and use of pigments in art.

We have demonstrated the potential and importance of noninvasive and label-free, multimodal chemical imaging for the study of an 1,800-year-old painting and one of the oldest surviving encaustic paintings in the world. The unique capacity to map molecular and elemental spectroscopic data of both organic and inorganic materials at the macroscopic scale, enhanced the determination of the raw materials used and the *chaîne opératoire* for the construction of this archaeologically significant painting. As materials demonstrate an invariant range of properties that determine their function, this portrait, through its material nature, helped reveal social practices and the beliefs and approaches of the Greco-Roman culture in their management of the physical world.

Multimodal imaging spectroscopy holds great promise for rapid data capture allowing the exploration of complex heterogeneous systems beyond art and archaeology, where noninvasive macroscale high-resolution mapping data is necessary for diagnostic or monitoring purposes.

## Methods

Hyperspectral reflectance and luminescence data cubes were collected with optimized pushbroom line-scanning imaging spectrometers. The first camera, operating from 400 to 1000 nm, consists of a transmission grating imaging spectrometer (N10E, Specim Corp., Finland) and a high sensitivity EMCCD detector array (ProEM: 1024B, Princeton Instruments, NJ) and was constructed by Surface Optics, CA. This line scanning camera has 2.5 nm sampling and operates at a light level of 1200 lux with integration time of 250 ms per line, providing signal to noise of 400:1 for a 99% diffuse white reflector. The infrared hyperspectral camera operating from 1000 to 2500 nm was built in house and utilizes a transmission grating imaging spectrometer (N25E, Specim Corp., Finland), a relay lens (Stingray Optics, NH) with external exit pupil, and a cryo-cooled 1280 by 1024 pixel InSb detector array (IRC912, IRCameras, CA). The detector is band-pass limited to light between 1000 and 2450 nm and has 100% cold stop efficiency. This line-scanning camera system has 2.8 nm sampling and operates at a light level of 770 lux with integration time of 150 ms per line, providing signal to noise >700:1 for a 99% diffuse reflector. Both the visible and infrared cameras allow for simultaneous collection of 1024 spectra along the spectrometer slit, giving a spatial sampling of 0.25 mm/pixel. The data cubes from both cameras were dark-corrected, flat-fielded, and calibrated to apparent reflectance using diffuse reflectance standards (Labsphere Inc, NH). The luminescence data cubes were corrected for self-absorption using the excitation profile and the reflectance data cube^54^. Once calibrated, all three data cubes were spatially registered to a reference color image using a novel algorithm^12^. The registration algorithm uses a polynomial fitting to transform the hyperspectral images such that they spatially align to the reference color image. The control point pairs used to fit the polynomial are constrained to have a disparity less than or equal to 1/3 of a pixel.

Identification of spectral endmembers and construction of maps from reflectance and luminescence data cubes were done using the hourglass method (ENVI, Harris, CO). The multivariate analysis has three key steps. First, principal components (PC) analysis is used to reduce the data dimensionality from that of the number of spectral bands to a much smaller number of principal component images. For example, for analysis of the pigments, the 230 spectral band images from 400–950 nm were used, and principal component analysis reduced the 230 spectral band images to 13 PC images. For the binder media, the determination of spectral endmembers was done using the 2230 to 2340 nm region after the spectral continuum was removed. Second, a convex geometry algorithm known as the Purity Pixel Index was used to find a subset of pixels in the hyperspectral cube whose spectra are the most unique and diverse. Finally, these pixels were then clustered in the reduced multidimensional space defined by the number of PC images that were retained. The average spectra of each cluster represent the ‘spectral endmembers’, which are used to make false-color maps using the spectral angle mapping algorithm, which identifies pixels in the hyperspectral cube whose reflectance spectra match the endmember spectra within a specified tolerance angle. Luminescence endmember determination and mapping were done using the same methods as for the pigment analysis except the luminescence cube was corrected for self-absorption using a modified model^55^.

Site-specific fiber optics reflectance spectra (FORS) from 350 to 2500 nm, 1.4 to 2 nm spectral sampling, and a spatial spot of 3 mm were used to verify the hyperspectral reflectance spectra. The instrument used was a fiber-optic spectroradiometer (FieldSpec 3, PANalytical-ASD Inc., CO).

MA-XRF data cubes were collected with a scanner designed in house using a rhodium X-ray source operating at 50 kV and 0.75 mA (XOS, NY) with converging polycapillary optics and a silicon drift detector (Vortex-90EX, Hitachi High- Technologies Science America, Inc.). The source was oriented normal to the painting surface and the detector was at ~45 degrees to the painting surface. The scanning was done with a high precision 2-axis easel (SmartDrive, UK). The scan rate gave a spatial sampling of 0.5 mm/pixel with 100% sampling efficiency. Integration time was 100 ms/pixel for a total scan time of 173 min, in contrast to less than 6 min total collection time for the hyperspectral reflectance scans. Each XRF spectrum of the MA-XRF image cube was fitted using a least square linear model^56^. Briefly, a Gaussian peak was fit to each detected emission line. Since the emission energies are well known, a Gaussian peak was fit if intensity was detected at the known energies using an offset (representing the local baseline) and amplitude characterized by the detector spectral response function at each emission energy. The resulting elemental maps display the integrated area under the labeled emission peaks. The maps are linearly stretched to display the minimum and maximum intensity values.
